# Precise optical modeling of quantum dots for white light-emitting diodes

**DOI:** 10.1038/s41598-017-16966-2

**Published:** 2017-11-30

**Authors:** Bin Xie, Yanhua Cheng, Junjie Hao, Weicheng Shu, Kai Wang, Xiaobing Luo

**Affiliations:** 1 School of Energy and Power Engineering, Huazhong University of Science and Technology, Wuhan, 430074 China; 2 Department of Electrical and Electronic Engineering, Southern University of Science and Technology, Shenzhen, 518055 China

## Abstract

Quantum dots (QDs)-based white light-emitting diodes (QDs-WLEDs) have been attracting numerous attentions in lighting and flat panel display applications, by virtue of their high luminous efficacy and excellent color rendering ability. However, QDs’ key optical parameters including scattering, absorption and anisotropy coefficients for optical modeling are still unclear, which are severely against the design and optimization of QDs-WLEDs. In this work, we proposed a new precise optical modeling approach towards QDs. Optical properties of QDs-polymer film were obtained for the first time, by combining double integrating sphere (DIS) system measurement with inverse adding doubling (IAD) algorithm calculation. The measured results show that the typical scattering, absorption and anisotropy coefficients of red emissive QDs are 2.9382 mm^−1^, 3.7000 mm^−1^ and 0.4918 for blue light, respectively, and 1.2490 mm^−1^, 0.6062 mm^−1^ and 0.5038 for red light, respectively. A Monte-Carlo ray-tracing model was set-up for validation. With a maximum deviation of 1.16%, the simulated values quantitatively agree with the experimental results. Therefore, our approach provides an effective way for optical properties measurement and precise optical modeling of QDs for QDs-WLEDs.

## Introduction

Colloidal quantum dots (QDs), as emerging nanocrystals light conversion materials, have been attracted more and more attentions in displays and lighting^1–3^. Unlike the bulk semiconductor material that has a fixed energy gap, the size range of QDs corresponds to the regime of quantum confinement for which electronic excitations feel the presence of the particle boundaries and respond to changes in the particle size by adjusting their energy spectra. This phenomenon is known as the quantum size effect. Quantum confinement leads to a collapse of the continuous energy bands of the bulk material into discrete ‘atomic’ energy levels. These well-separated QDs states can be labeled using atomic-like notations (1S, 1P, 1D, etc). Due to the strong quantum confinement in three dimensions, QDs’ emission spectra can be easily tuned from ultraviolet to infrared by changing their particle size and chemical composition, and their full width at half maximum (FWHM) is much narrower than that of conventional phosphor, leading to precise spectral control^4–7^. QDs can be excited by both photoluminescence and electroluminescence^8–10^. With these extraordinary characteristics, QDs-based white light-emitting diodes (QDs-WLEDs) possess high luminous efficacy as well as high color rendering ability^11–13^, making them promising candidates for next-generation solid-state lighting (SSL).

**Figure 1:**
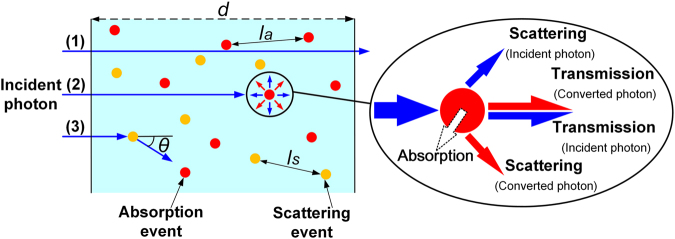
Schematic showing all the possible behaviors between incident blue photons and the QDs layer.

In one QDs-WLED, blue light is firstly emitted from GaN LED chip by means of electroluminescence (EL), and then irradiate on light conversion materials (e.g. phosphor, QDs). The mixture of transmitted blue light and long-wavelength light converted by QDs produces white light. Figure 1 shows the schematic of all the possible behaviors between the incident blue photons and the QDs. The blue photons may either be scattered by QDs (light path (3)), resulting in a change of their movement direction, or be absorbed by QDs (light path (2)), and their energy is converted into QDs emission light or transformed into heat energy. Besides, some of the blue photons may even pass the QDs layer without been affected at all (light path (1)). Moreover, photons emitted from QDs also will have the chance to be scattered by other QDs during transmission (Inset). Therefore, photons’ behaviors, including absorbing, remitting, scattering and transmitting, are very complicated in QDs-WLED, and it is necessary and critical to build up an optical model of QDs-WLED for further device optimization.

Generally, three key optical parameters, absorption coefficient, scattering coefficient and anisotropy coefficient, are utilized to characterize the behaviors of light absorption, conversion, scattering, and propagation in the QDs layer. Previous studies have presented some optical models of QDs to simulate their optical performances in various optoelectronic devices^14–17^. In an approach, QDs are treated as dipoles and transfer matrix formalism^11^ is utilized to calculate the light out coupling and angular performance of QDs-based device^14,15^. While there are difficulties in directly measuring the QDs’ microcosmic parameters such as power dissipation density, wave vector and intrinsic quantum yield *et al*., making this method impractical to simulate real QDs-polymer film. In another approach, Monte-Carlo ray-trace method is performed to theoretically evaluate the performance of QDs-based materials^16,17^. Unlike the first method, this approach is conducted by inputting the absorption, PL spectra of QDs, the attenuation coefficient and QY of QDs, which are easier to be measured and are practical to QDs devices. In previous literatures, before processing the Monte-Carlo ray-trace simulation, some assumptions have to be made to conduct the calculation, including assuming that light scattering won’t occur in the QDs-polymer film due to the small size of QDs^16,18,19^. However, as far as we are concerned, this assumption is not always reasonable because the scattering coefficient of QDs-polymer film is non-negligible, especially when scattering particles are doped in^20–23^. Therefore, a more precise optical model of QDs, including coefficients of absorption, scattering and anisotropy, need to be developed for QDs-WLED design and optimization.

Here, we reported on a precise optical model of QDs by combining double integrating spheres (DIS) system with inverse adding-doubling (IAD) algorithm. The proposed model takes into account all the fate of incident photons, including transmittance, absorption and scattering, which is compatible with the real situation. A series of QDs-polymer films samples with varying QDs concentrations were fabricated, and their transmittance, reflectance and collimating transmittance were measured by the DIS system. Then their optical properties of scattering coefficient, absorption coefficient and anisotropy coefficient were calculated by the IAD algorithm. Finally, these parameters were adopted in Monte-Carlo ray-tracing model to conduct the simulation, and the ray-tracing results were compared with the experimental results to validate the feasibility of our model.

## Experimental setup

### Definitions

As depicted in Fig. 1, the optical properties of QDs layer can be characterized by quantities as follows:

1 $${u}_{s}=1/{I}_{s},$$

2 $${u}_{a}=1/{I}_{a}.$$

where the scattering coefficient *µ*_s_ and the absorption coefficient *µ*_a_ are defined as the reciprocal of the average free path between two scattering events (*I*_s_) or two absorption events (*I*_a_), respectively. If the QDs layer is highly absorbing, the number of absorption events will be big, leading to a small average free path *I*_a_, which yields a high absorption coefficient *µ*_a_. Therefore, the higher the scattering or the absorption of a QDs layer is, the higher the respective coefficient would become.

In addition to the above two optical quantities, there is another parameter, single-scattering phase function *p*(*θ*), which characterizes the amount of light scattered at an angle *θ* from the incoming direction. It is often expressed in terms of the cosine of the scattering angle *θ*:

3 $${\int }_{4\pi }p(v)d\omega =2\pi {\int }_{-1}^{1}p(v)dv=1,\,v=\,\cos \,\theta .$$

where *d*ω is a differential solid angle, and *v* is the cosine of the scattering angle. The functional form of *p*(*v*) is usually unknown. While Jacques *et al*.^24^ and Yoon *et al*.^25^ have demonstrated that Henyey-Greenstein function approximates single-particle light scattering in human dermis and aorta at 633 nm. Therefore, this phase function is adopted in our calculation:

4 $$p(v)=\frac{1}{4\pi }\cdot \frac{1-{g}^{2}}{{(1+{g}^{2}-2gv)}^{3/2}}.$$

The Henyey-Greenstein phase function depends on the anisotropy coefficient *g*, which is defined as:

5 $$g={\int }_{4\pi }p(v)vd\omega =2\pi {\int }_{-1}^{1}p(v)vdv.$$

The anisotropy coefficient *g* ranges from −1 to 1. When *g* = −1, scattering is all directed into reverse direction, when *g* = 1, scattering is all directed into forwarding direction, and when *g* = 0, scattering is equally probable in all directions. Using the physical sample thickness *d*, the coefficients *µ*_s_ and µa can be expressed by two dimensionless quantities, the albedo *a* and the optical thickness *τ* ^26^:

6 $$a=\frac{{\mu }_{s}}{{\mu }_{s}+{\mu }_{a}}=\frac{{\mu }_{s}d}{\tau },$$

7 $$\tau =d\cdot ({\mu }_{s}+{\mu }_{a}).$$

The albedo varies between 0 and 1, *a* = 0 indicates that no scattering occurs in the sample, while *a* = 1 indicates the absence of absorption. The optical thickness is defined as the product of the physical sample thickness and the sum of the scattering and the absorption coefficient. For a sample with the optical thickness *τ* = 1, there is a probability of *e* ^−1^ = 37% that light will travel through it without being scattered or absorbed. In the following contents, the optical properties will be calculated in terms of three dimensionless quantities *a*, *τ*, and *g*.

### Measurement setup

**Figure 2:**
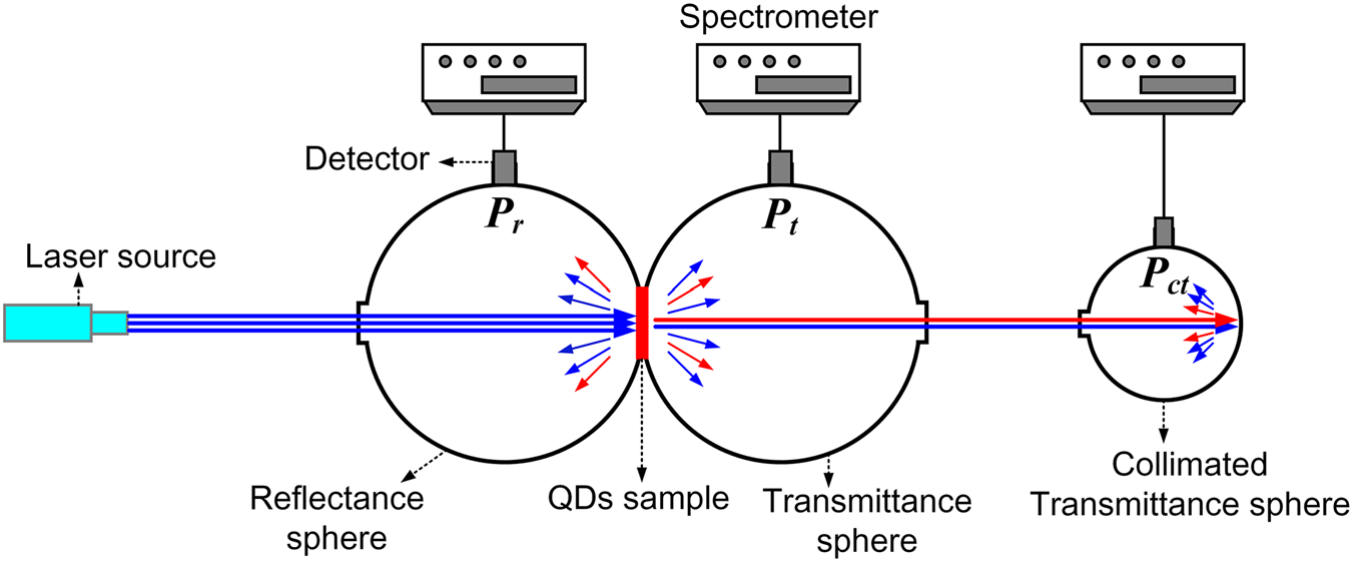
Schematic of set-up of a double integrating sphere system.

The measurement of the optical properties of QDs samples can be achieved by the use of a double integrating sphere set-up, which is schematically depicted in Fig. 2. The QDs sample is placed between two integrating spheres so that it is situated at the exit port of the reflectance sphere (used to measure the reflectance *P*_r_), and on the entrance port of the transmittance sphere (used to measure the transmittance *P*_t_). These two ports have identical sizes. Besides, the third integrating sphere (collimating transmittance sphere) is situated directly behind the transmittance sphere and is used to measure the light power *P*_ct_ that is specularly transmitted through the sample. The entering light is incident perpendicularly upon the sample.

To measure the reflectance and the transmittance of a sample, the photomultiplier is usually used as detectors^27^. Theoretically, the voltage *V*_δ_ recorded by a detector is proportional to the light power *P*_δ_ incident upon it:

8 $${V}_{\delta }=K{P}_{\delta }.$$

where *K* is the constant proportionality, which depends on the detector characteristics. Due to the background signal *V*_0_, which occurs in all measurements and originates from noise in the system and the presence of stray light, expression (9) has to be expanded as follows:

9 $${V}_{\delta }=K{P}_{\delta }+{V}_{0}.$$

Therefore, the detected power can be expressed as:

10 $${P}_{\delta }=\frac{1}{K}({V}_{\delta }-{V}_{0}).$$

The constant *K* can be eliminated by using reference measurements which conducted with a single integrating sphere set-up. For a reference plate in the sample position, *P*_ref_ can be determined by:

11 $${P}_{ref}=\frac{1}{K}({V}_{ref}-{V}_{ref,0}).$$

Therefore, by conducting the reference measurements, the relative values *V*_r_%, *V*_t_% and *V*_ct_% can be calculated as:

12 $${V}_{r} \% =\frac{{V}_{r}-{V}_{r,0}}{{V}_{ref}-{V}_{ref,0}};{V}_{t} \% =\frac{{V}_{t}-{V}_{t,0}}{{V}_{ref}-{V}_{ref,0}};{V}_{ct} \% =\frac{{V}_{ct}-{V}_{ct,0}}{{V}_{ref}-{V}_{ref,0}}.$$

where the values *V*_r,0_, *V*_t,0_ and *V*_ct,0_ represent the background signals corresponding to the respective measurements *V*_r_, *V*_t_ and *V*_ct_.

### Calculation theory

As mentioned above, the final aim is to obtain the optical properties given by *a*, *τ*, and *g*, respectively. While the DIS measurements do not yield *a*, *τ*, and *g* directly, several measurement values that depend on the reflectance and the transmittance of the sample. Therefore, we have to find a connection between the optical properties and the measurement results. This can be done by the use of an appropriate model of radiative transfer. One of these models is used in the Adding Doubling (AD) Method^27^, which was introduced by van de Hulst as one-dimensional numerical solution of the radiative transport equation. For given optical properties (albedo *a*, optical thickness *τ*, and anisotropy coefficient *g*), the reflection factors (*R*_d_, *R*_cd_, *R*_c_) and the transmission factors (*T*_d_, *T*_cd_, *T*_c_) can be calculated by using this algorithm:

13 $${f}_{AD}:(a,\tau ,g)\to ({R}_{d},{R}_{cd},{R}_{c},{T}_{d},{T}_{cd},{T}_{c}).$$

where the reflection factor *R* and the transmission factor *T* are defined relative to the irradiance on the sample surface. They vary between 0 and 1 and denote the fraction of the total incident light that is reflected or transmitted by the sample, respectively. For diffuse light incident upon the sample, the diffuse reflection (transmission) factor *R*_d_ (*T*_d_) denotes the fraction of light that is reflected (transmitted) diffusely by the sample. For collimated light incident upon the sample, the diffuse reflection (transmission) factor *R*_cd_ (*T*_cd_) denotes the fraction of light that is reflected (transmitted) diffusely by the sample. The collimated reflection (transmission) factor *R*_c_ (*T*_c_) denotes the fraction of light that is reflected (transmitted) specularly.

The AD method assumes that the reflection function *R*(*ν*, *ν*’) and transmission function *T*(*ν*, *ν*’) for light incident at an angle *ν* and exiting at an angle *ν*’ are known for one layer. For two similar and adjacent slabs, the light traveling from left to right across the boundaries 0, 1 and 2. The reflection and transmission of the two slabs can be derived from the results of the thin slab by using the AD equations^28,29^:

14 $${R}^{02}={R}^{20}=T{(E-R\ast R)}^{-1}R\ast T+R,$$

15 $${T}^{02}={T}^{20}=T{(E-R\ast R)}^{-1}T.$$

where *R*_01_ and *T*_01_ are defined as the reflection and transmission operators for light incident upon boundary 0 and moving towards boundary 1, respectively, and vice-versa. *E* is the unity matrix. Beginning with the starting thickness *τ*_start_, *R*_start_ and *T*_start_, this doubling process repeatedly doubles the slab thickness by using the expressions (14) and (15), until the desired slab thickness *τ* has been reached. *τ*_start_ has to be far smaller than the thickness of QDs sample d (*τ*_start_ = 2^−*n*^d, *n* = 100~1000). After decided the *τ*_start_, the *R*_start_ and *T*_start_ can be determined by the initialization routine^29^. The resulting *R* and *T* are taken as the reflection matrix and the transmission matrix of the sample.

The correlation between the measurement data and the reflection/transmission factor can be calculated by the ‘double integrating sphere function’:

16 $${f}_{DIS}:({R}_{d},{R}_{cd},{R}_{c},{T}_{d},{T}_{cd},{T}_{c})\to ({V}_{r} \% ,{V}_{t} \% ,{V}_{ct} \% ).$$

With the expressions (13) and (16), we can deduce that, the measurement results *V*_r_%, *V*_t_% and *V*_ct_% can be predicted, if the optical properties *a*, *τ* and *g* of the sample are given, by a combination of the functions *f*_AD_ and *f*_DIS_:

17 $${f}_{DIS}\cdot {f}_{AD}:(a,\tau ,g)\to ({V}_{r} \% ,{V}_{t} \% ,{V}_{ct} \% ).$$

In our case, we want to perform the calculation in the other direction. An iterative solution of the problem is given by the Inverse Adding Doubling (IAD) method, the function can be expressed as:

18 $${f}_{IAD}={({f}_{DIS}\cdot {f}_{AD})}^{-1}:({V}_{r} \% ,{V}_{t} \% ,{V}_{ct} \% )\to (a,\tau ,g).$$

**Figure 3:**
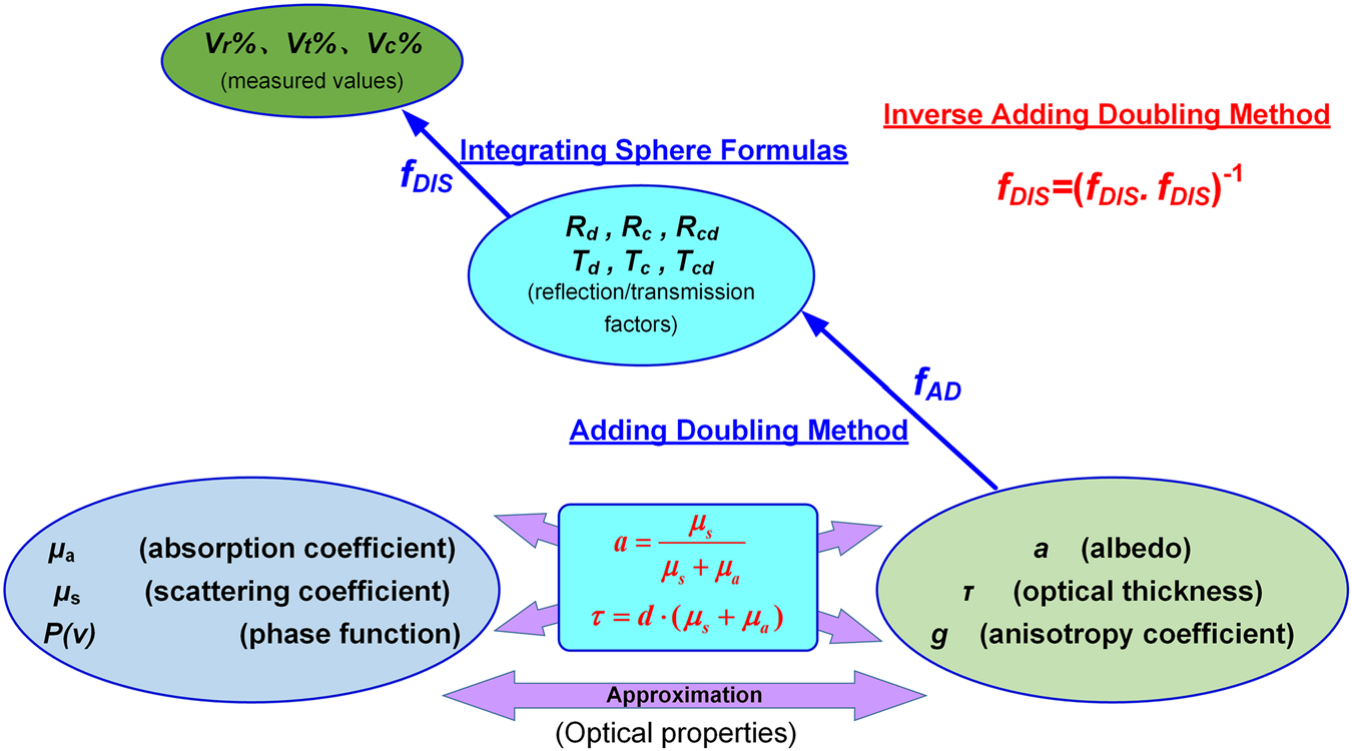
Correlations between the three levels of quantities.

Figure 3 shows the correlations between the three levels of quantities. Consequently, the procedure to obtain the optical properties of a sample by the use of DIS set-up and IAD algorithm is as follows:

1. Conduct the reference measurements to calibrate the measurement set-up.
2. Determine the three measurement values *V*_r_%, *V*_t_% and *V*_ct_% for the sample.
3. Calculate the optical properties *a*, *τ* and *g* with the IAD algorithm, by using the measurement data as the input of the program.
4. Calculate *µ*_a_ and *µ*_s_ from the returned values *a* and *τ*.

## Results and Discussion

To verify the accuracy and effectiveness of our IAD algorithm, we performed an AD calculation for an ‘imaginary’ sample with the optical properties *a* = 0.8, *τ* = 1, *g* = 0.8 and a refractive index *n*_s_ = 1.5. The sample was situated between two ‘imaginary’ glasses with reflection factor *R*_g1_ = *R*_g2_ = 0.04 and transmission factor *T*_g1_ = *T*_g2_ = 0.96 (*n*_g_ = *n*_s_ = 1.5). By using the AD function described in expression (13), we can get the ‘measurement values’

19 $${V}_{r} \% =0.0454;\,\,\,\,\,\,{V}_{t} \% =0.3039;\,\,\,\,\,\,{V}_{ct} \% =0.33911.$$

**Figure 4:**
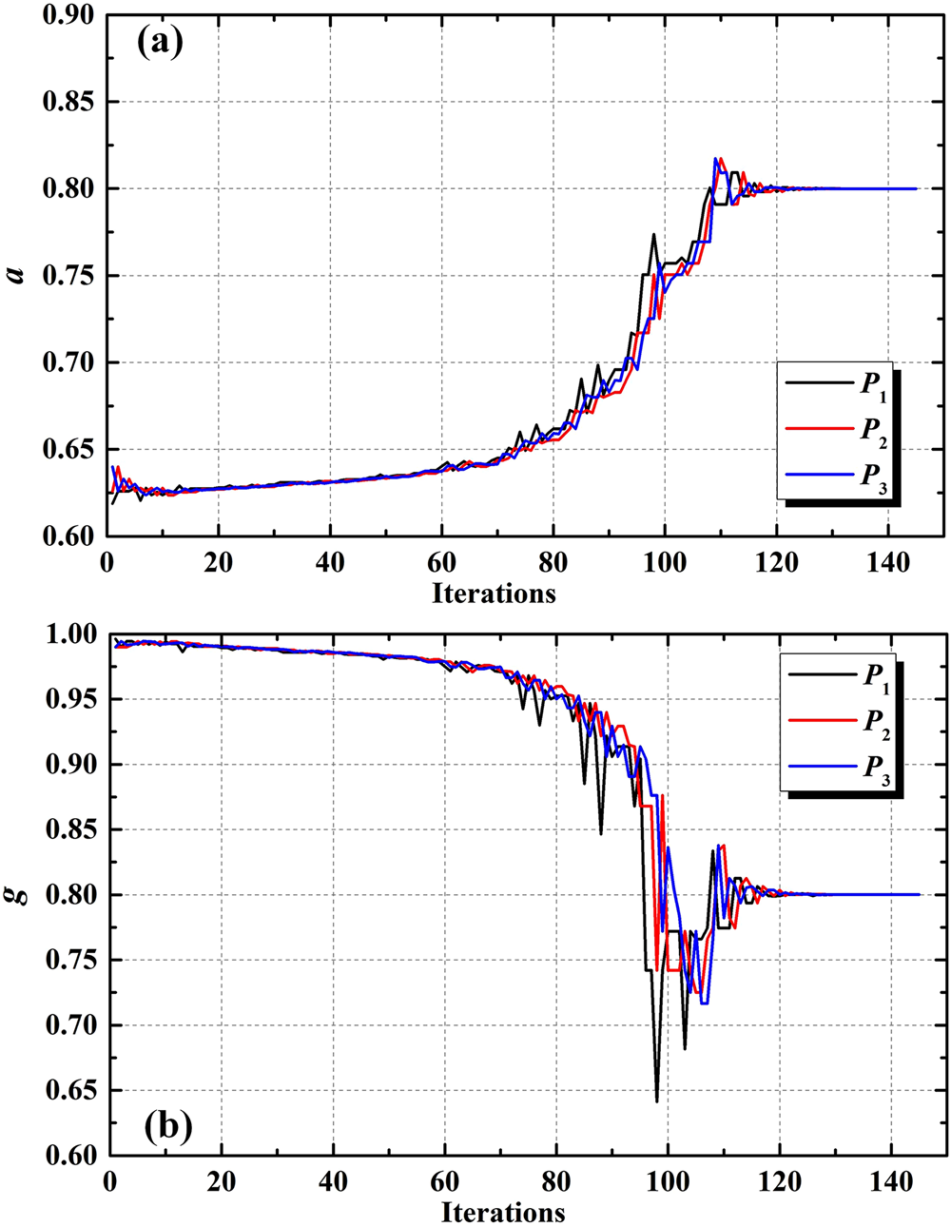
Development of (**a**) *a* and (**b**) *g* during the iteration.

In another direction, if we use above three values in equation (19) as the inputs for the IAD algorithm, we should obtain the optical properties *a* = 0.8, *τ* = 1, *g* = 0.8 as a result. As all three measurement values are known, all optical properties can be determined. Figure 4 shows the development of *a* and *g* during the iteration. In our IAD program, the routine terminates after 145 iteration steps, returning the values of *a* = 0.7999 and *g* = 0.8001. Therefore, our IAD algorithm has high accuracy and fast convergence speed.

Without loss of generality, CdSe/ZnS core/shell QDs were used as the QDs-polymer film samples to validate our model. High-quality red-emissive QDs with an optimized peak wavelength of 626 nm were prepared by the Tri-n-Octylphosphine (TOP)-assisted successive ionic layer adsorption and reaction (SILAR) method^11,30^. According to our previous work^31^, adding QDs with a peak wavelength of 626 nm into phosphor-converted LEDs can greatly improve the color rendering index. Polymethyl methacrylate (PMMA) was chosen as the polymer matrix of QDs film samples. The QDs-PMMA film was prepared with the *in-situ* polymerization method^32^. Firstly 25 mL of MMA monomer and 0.2% wt/wt of azodiisobutyronitrile (AIBN) with respect to MMA were mixed in a three-neck flask and kept stirring until the AIBN was dissolved completely. Then 3 mL of MMA-AIBN solution was transfer to a cleaned centrifuge tube and 10 µL of QDs solution was added into the tube, and the mixture was homogeneously dispersed by the ultrasound treatment. After that, the tube was placed into the thermostatic water bath at 70 °C for 15 min until the mixture reached certain viscosity, and then cooled to temperature. Then, the viscous liquid was introduced into the tailored mould. The mould was placed in the vacuum oven at 45 °C and kept for 24 h. Finally, the resulting film was cut into circle and labeled as sample No. 1. Similarly, Nos 2–6 samples were prepared by adding 20, 30, 40, 50 and 60 µL of QDs solution into 3 mL of MMA-AIBN solution.

**Figure 5:**
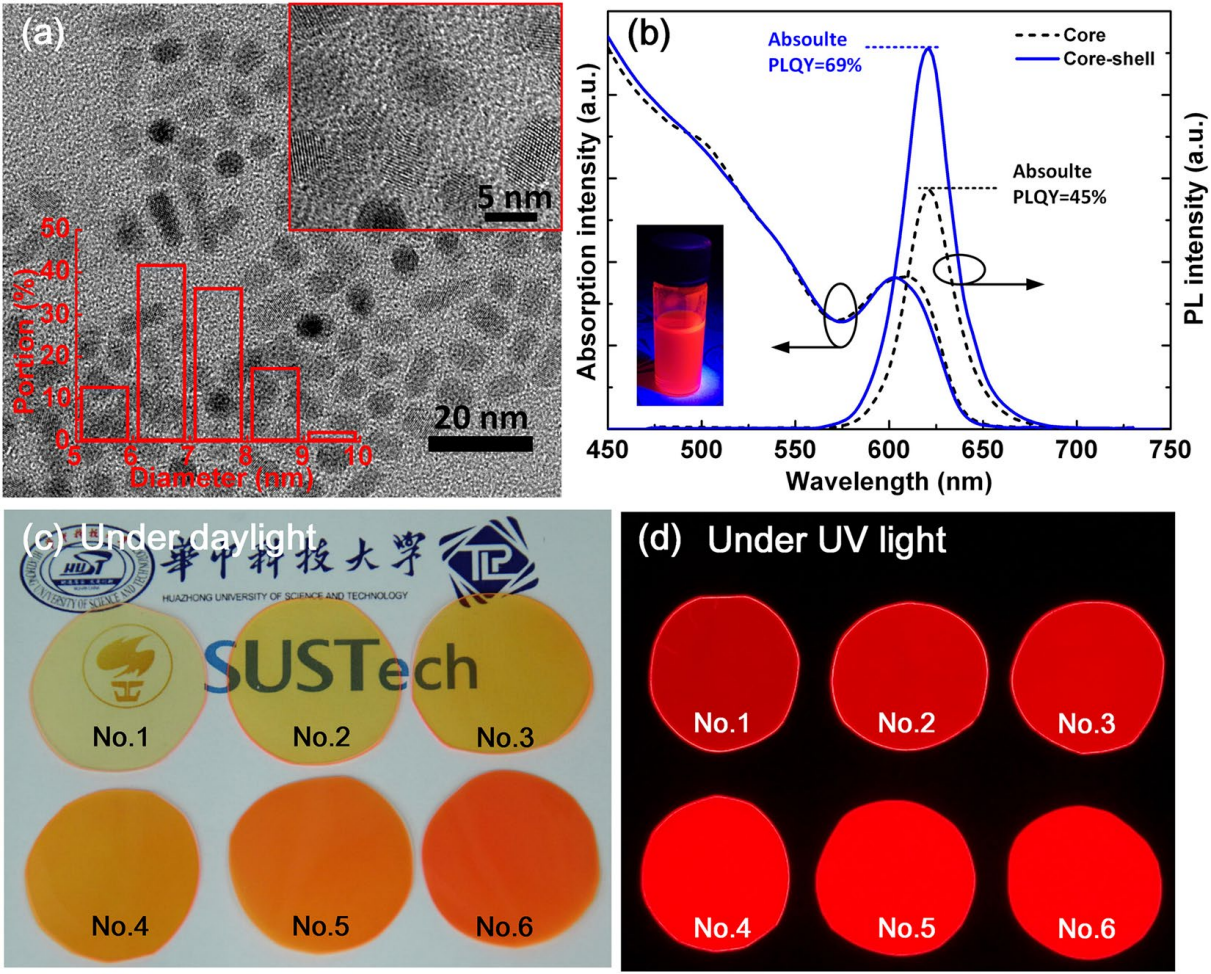
(**a**) HRTEM images of the as-prepared CdSe/ZnS core-shell QDs. (**b**) Absorption and PL spectra of the CdSe core and CdSe/ZnS core-shell QDs. The inset shows the CdSe/ZnS QDs solution under UV light exposure. (**c**) and (**d**) show the photographs of the as-prepared QDs-PMMA film samples under daylight and UV light, respectively.

Figure 5(a) shows the high-resolution transmission electron microscope (HRTEM) images of the as-prepared QDs, from which the average diameter of QDs is measured as 6.8 nm with uniform size distribution. Figure 5(b) depicts the absorption and PL spectra of the CdSe core and CdSe/ZnS core-shell QDs. The narrow width at half maximum (FWHM) of near 31 nm from PL spectra has confirmed their uniform size distribution. Due to the TOP-assisted SILAR method, which removes the surface lattice imperfections by the surface ions re-dissolution and lattice re-arrangement during the whole ZnS shell formation process, the absolute PL quantum yields (PLQY) was enhanced from 45% to 69%. Figure 5(c) and (d) show the photographs of the QDs-PMMA film under daylight and UV light, respectively. The as-prepared film samples maintain high transparency and uniformity, and the thickness of these films are measured as 0.41 to 0.44 mm.

In QDs-LEDs packaging, the main concerns are the absorption and scattering properties of QDs film for blue light emitted from the LED chip and converted red light emitted from itself. Therefore, in the DIS measurement, two diode lasers with peak wavelength of 450 nm and 650 nm were used as the light sources. The optical power of these lasers is 20 mW, and their beam diameter is expanded to 8 mm to illuminate a sufficiently large area of QDs-PMMA film. Diameters of the reflectance, transmittance and collimating transmittance spheres are 300 mm, 300 mm and 150 mm, respectively. Diameters of the apertures are 18 mm. For blue light (450 nm) excitation, the spectra of transmitted and reflected light are divided into two parts at 500 nm. Light with spectra from 380 nm to 500 nm is defined as unconverted blue light. For red light (650 nm) excitation, since no converted light is emitted from the QDs film, both the transmitted light and reflected light are sorted as the red light.

**Figure 6:**
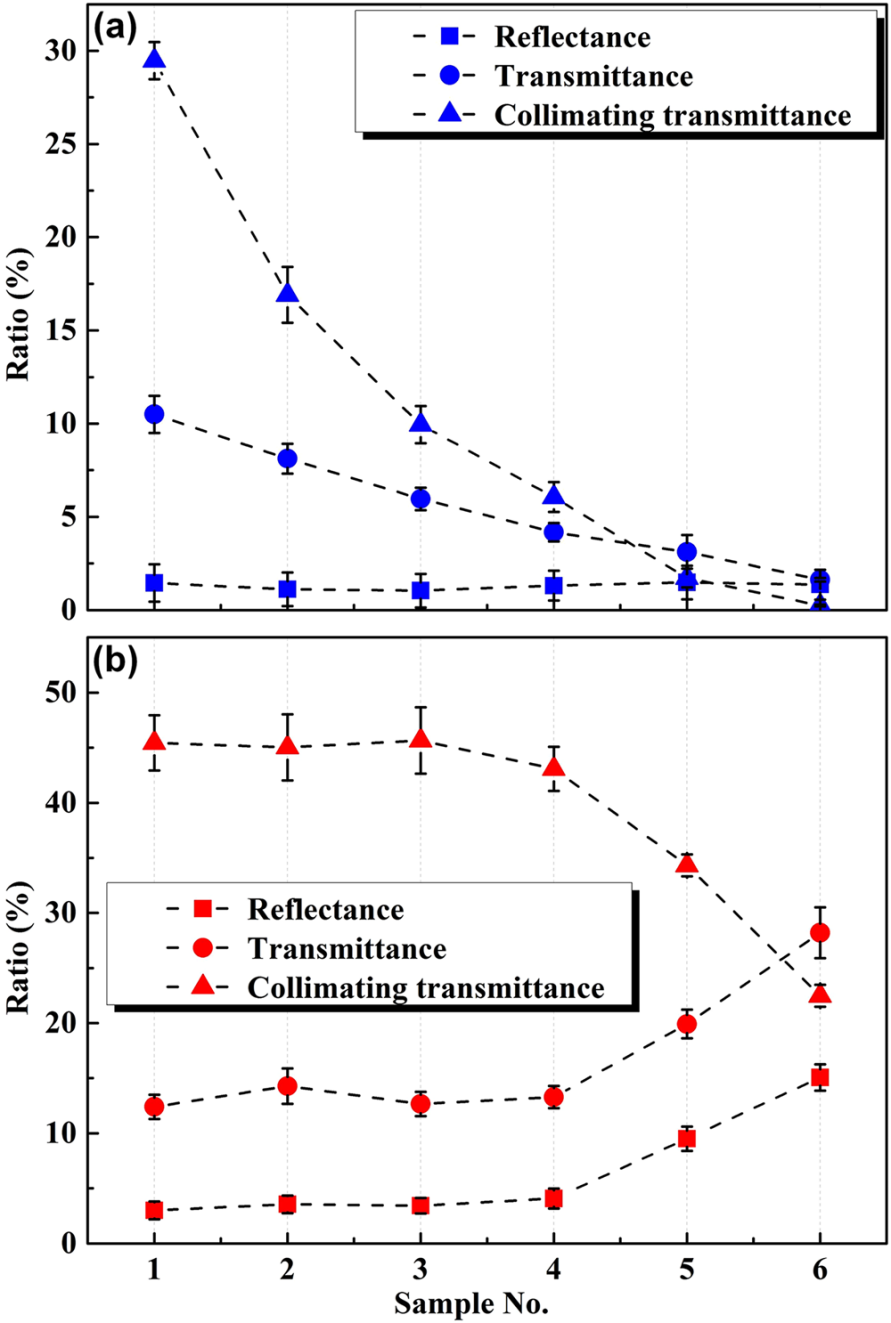
(**a**) Measured reflectance, transmittance and collimating transmittance of QDs-PMMA samples based on blue laser incidence. (**b**) Measured reflectance, transmittance and collimating transmittance of QDs-PMMA samples based on red laser incidence.

Figure 6 shows the measured reflectance and transmittance of QDs-PMMA samples based on blue and red light incidence. For simplicity, the reflectance, transmittance and collimating transmittance of the blue light and the red light are denoted as *V*_RB_, *V*_TB_, *V*_CTB_, *V*_RR_, *V*_TR_ and *V*_CTR_. In the case of blue light incidence, *V*_RB_ only takes a very small proportion (1.04%~1.45%), and it varies little against the QDs concentration. This is mainly because, during the light propagation, the backward scattered blue light is further absorbed by the QDs particles. It can be found that the QDs present remarkable high absorption and scattering for the blue light, and cause rapid decrease of *V*_CTB_ and small reduction of *V*_TB_ when the QDs concentration increased.

In the case of red light incidence, the results are quite different from those of blue light. The *V*_RR_ and *V*_TR_ increased with the increasing QDs concentration, which depicts that the increase of QDs particles leads to the enhancement of multiple scattering effects, and the backward scattered red light can emit out due to the weak absorption of the QDs. Consequently, the *V*_CTR_ decreased with the increasing QDs concentration, because more and more light changed propagation direction.

According to the measured reflectance and transmittance values, the optical properties (*µ*_s_, *µ*_a_ and *g*) of QDs-PMMA film samples are calculated by IAD algorithm, and the results are listed in Table 1. Measured results showed that the QDs present strong absorption for blue light, high reflection for converted red light, and an anisotropic emission pattern of converted red light. It is seen that the scattering and absorption coefficients increase with the increasing QDs concentration, while the anisotropy coefficient changes slightly with the QDs concentration. Under the same QDs concentration, the scattering and absorption coefficients toward blue light incidence are large than that of red light incidence, which agrees with the measured results.

**Table 1:** Calculated optical properties of QDs-PMMA film samples toward blue and red light incidence.

Sample No.	*µ*_s_ (mm^−1^)		*µ*_a_ (mm^−1^)		g	
	450 nm	650 nm	450 nm	650 nm	450 nm	650 nm
1	1.3772	1.0691	1.4042	0.6564	0.5830	0.5555
2	1.7171	1.1117	2.2259	0.5543	0.5933	0.5737
3	2.1682	1.0585	2.8922	0.5890	0.5600	0.5297
4	2.9382	1.2490	3.7000	0.6062	0.4918	0.5038
5	4.7513	1.8611	4.2921	0.3839	0.5586	0.4455
6	8.0145	2.8908	5.5025	0.3146	0.6551	0.4828

To validate our calculated optical properties of QDs film, an optical model of DIS system, which imitate the real DIS measurement system, was built up. The blue light (450 nm) is emitted out perpendicular to the laser aperture, with a beam divergence angle of 1.2 mrad. The inner surfaces of the three integrating spheres are coated with a diffuse white material having optical properties of 11.1% absorption and 89.9% scattering, and are used as the receivers to collect the transmitted and reflected light.

The Monte Carlo method is applied for ray-tracing of the optical model. Total number of the traced rays is 2 million, of which 1 million rays are the blue light and the other 1 million rays are the converted-red light. The threshold of light rays is 10–4. During the ray-tracing, the energy of light rays is calculated by optical power in watts. Light rays with single wavelength are used to represent the blue light and the converted-red light, which are set to be 450 nm and 626 nm, respectively. This simplification has been proven to be effective for the ray-tracing of light-emitting process^33–37^.

**Figure 7:**
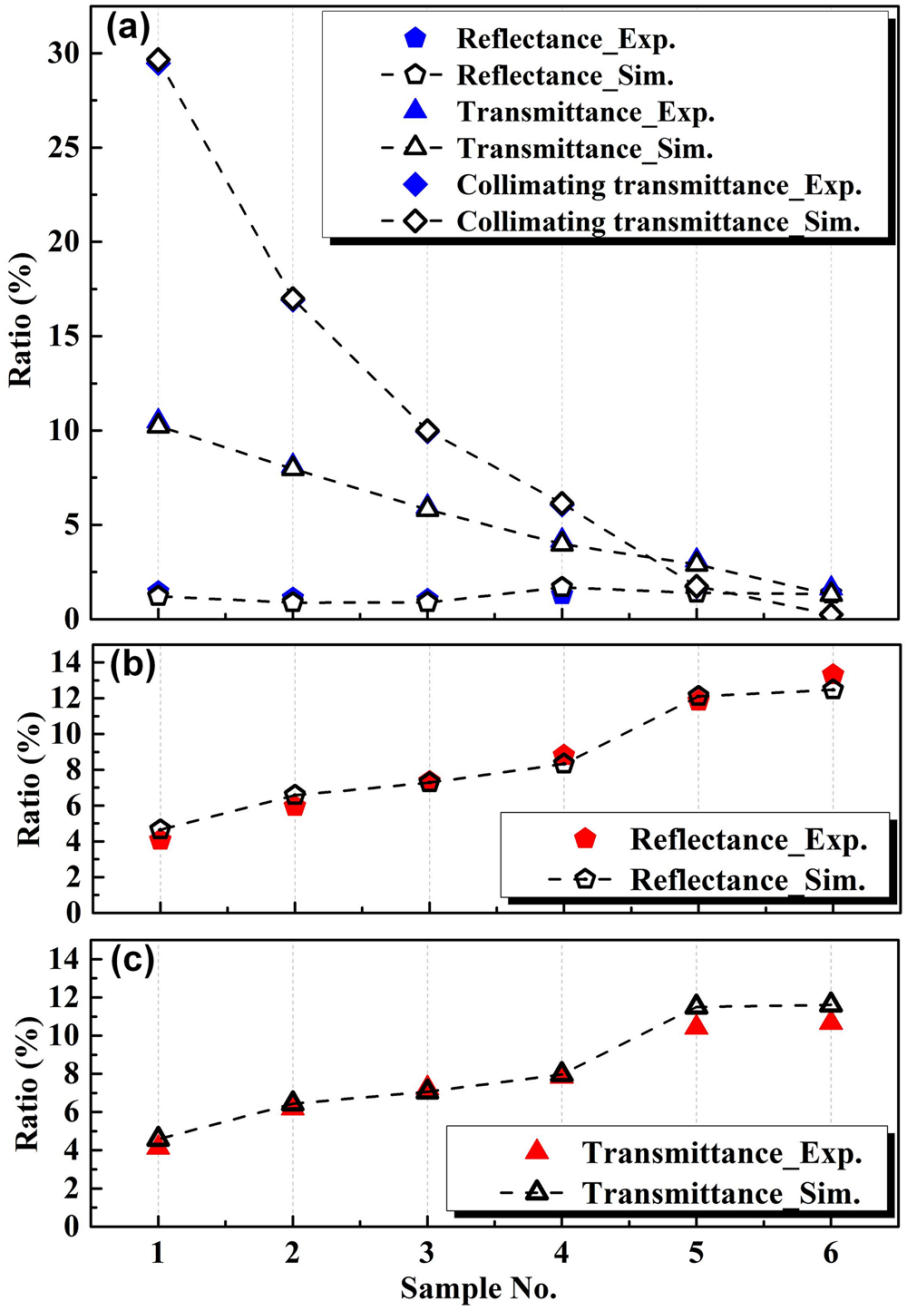
(**a**) Comparisons of simulated and experimental reflectance, transmittance and collimating transmittance of incident blue light. (**b**) Comparisons of simulated and experimental reflectance of converted red light. (**c**) Comparisons of simulated and experimental transmittance of converted red light.

Figure 7(a) shows the comparisons between the ray-tracing and experimental results of reflectance, transmittance and collimating transmittance for the incident blue light. It can be seen that the simulated *V*_TB_ and *V*_CTB_ values agree well with the experimental results, with a maximum deviation of 0.26%. The *V*_RB_ values in the ray-tracing are slightly larger than those in the experiments, with a maximum deviation of 0.32%, respectively. This larger difference is mainly due to the relatively low value of *V*_RB_. Figure 7(b) and (c) show the comparisons of those results for the converted red light. Note that the collimating transmittance values of the converted red light are not displayed here due to their weak signals that can hardly be collected by the photodetector. With a maximum deviation of 1.16%, the simulated values quantitatively agree with the experimental results. Therefore, the QDs optical properties can be measured and calculated precisely by our approach, which provides wide guidance for QDs-WLEDs design and fabrication.

## Conclusion

In summary, this work provides a precise optical modeling approach towards QDs-polymer materials. The optical properties of QDs-PMMA film were measured by a double integrating sphere system and calculated by the inverse adding doubling algorithm. Measured results showed that the QDs present strong absorption for blue light, high reflection for converted red light, and an anisotropic emission pattern for both blue light and converted red light. Based on the calculated results, the absorption coefficient, scattering coefficient, and anisotropy coefficient were imported into the Monte-Carlo ray-tracing model for validation. With a maximum deviation of 1.16%, the simulated values quantitatively agree with the experimental results. Therefore, our optical modeling approach can obtain the necessary optical properties of QDs-polymer film, and the parameters can be utilized to conduct the ray-tracing simulation of QDs-based devices.

### Data availability

All data generated or analyzed during this study are included in this published article.

## References

[1] Pimputkar S, Speck JS, DenBaars SP, Nakamura S (2009). Prospects for LED lighting. Nat. Photon..

[2] Luo X, Hu R, Liu S, Wang K (2016). Heat and fluid flow in high-power LED packaging and applications. Prog. Energ. Combust. Sci..

[3] Jang HS, Jeon DY (2007). White light emission from blue and near ultraviolet light-emitting diodes precoated with a Sr_3_SiO_5_:Ce^3+^,Li^+^ phosphor. Opt. Lett..

[4] Cho K-S (2009). High-performance crosslinked colloidal quantum-dot light-emitting diodes. Nat. Photononics..

[5] Luo Z, Chen H, Liu Y, Xu S, Wu S-T (2015). Color-tunable light emitting diodes based on quantum dot suspension. Appl. Opt..

[6] Chen K-J (2015). Efficient hybrid white light-emitting diodes by organic-inorganic materials at different CCT from 3000K to 9000K. Opt. Express..

[7] Xie B, Hu R, Luo X (2016). Quantum dots-converted light-emitting diodes packaging for lighting and display: status and perspectives. J. Electron. Packag..

[8] Li Q (2017). Solid ligand-assisted storage of air-stable formamidinium lead halide quantum dots via restraining the highly dynamic surface toward brightly luminescent light-emitting diodes. ACS Photonics..

[9] Li F (2017). Quantum dot white light emitting diodes with high scotopic/photopic ratios. Opt. Express..

[10] Zhang Q (2017). Enhancing extraction efficiency of quantum dot light-emitting diodes by surface engineering. Opt. Express..

[11] Chen W (2016). High efficiency and color rendering quantum dots white light-emitting diodes optimized by luminescent microspheres incorporating. Nanophotonics..

[12] Erdem T, Nizamoglu S, Sun XW, Demir HV (2010). A photometric investigation of ultra-efficient LEDs with high color rendering index and high luminous efficiency employing nanocrystal quantum dot luminophores. Opt. Express..

[13] Zhong P, He G, Zhang M (2012). Optimal spectra of white light-emitting diodes using quantum dot nanophosphors. Opt. Express..

[14] Zhang X, Hagglund C, Johansson EMJ (2016). Electro-optics of colloidal quantum dots solids for thin-film solar cells. Adv. Func. Mater..

[15] Zhu R, Luo Z, Wu S-T (2014). Light extraction analysis and enhancement in a quantum dot light emitting diode. Opt. Express..

[16] Hu X (2015). Ray-trace simulation of CuInS(Se)(2) quantum dot based luminescent solar concentrators. Opt. Express..

[17] Knowles KE (2015). Bright CuInS_2_/CdS nanocrystal phosphors for high-gain full-spectrum luminescent solar concentrators. Chem. Comm..

[18] Kennedy, M. Monte-Carlo Ray-trace Modelling of Quantum Dot Solar Concentrator (Dublin Institute of Technology, 2010).

[19] Şahin D, Ilan B, Kelley DF (2011). Monte-Carlo simulations of light propagation in luminescent solar concentrators based on semiconductor nanoparticles. J. Appl. Phys..

[20] Zhu Y (2016). Light conversion efficiency enhancement of modified quantum dot films integrated with micro SiO_2_ particles. J. Disp. Tech..

[21] Yang J (2012). Electrospun TiO2 microspheres as a scattering layer for CdS quantum dot-sensitized solar cells. J. Electroanal. Chem..

[22] Xu X (2012). Mesoporous titania hollow spheres applied as scattering layers in quantum dots sensitized solar cells. Mater. Chem. Phys..

[23] Samadpour M, Zad AI, Molaei M (2014). Simply synthesized TiO_2_ nanorods as an effective scattering layer for quantum dot sensitized solar cells. Chin. Phys. B..

[24] Jacques SL, Alter CA, Prahl SA (1987). Angular dependence of HeNe laser light scattering by human dermis. Lasers Life Sci..

[25] Yoon G, Welch AJ, Motamedi M, Gemert MCJV (1987). Development and application of 3-dimensional light distribution model for laser irradiated tissue. IEEE J. Quantum Electron..

[26] Prahl SA, van Gemert MJ, Welch AJ (1993). Determining the optical properties of turbid media by using the adding-doubling method. Appl. Opt..

[27] van de Hulst, H. C. Multiple light scattering: tables, formulas, and applications (Elsevier, 1980).

[28] Prahl, S. A. The adding-doubling method in *Optical thermal response of laser irradiated tissue* (eds Welch, A. J. & van Gemert, M. J. C.) 101–129 (Springer, 1995).

[29] Poppendieck, W. Double integrating spheres: A method for assessment of optical properties of biological tissues (Institutionen för medicinsk teknik, 2004).

[30] Hao J, Zhou J, Zhang C (2013). A tri-n-octylphosphine-assisted successive ionic layer adsorption and reaction method to synthesize multilayered core-shell CdSe-ZnS quantum dots with extremely high quantum yield. Chem. Comm..

[31] Xie B (2017). Realization of wide circadian variability by quantum dots-luminescent mesoporous silica-based white light-emitting diodes. Nanotechnology..

[32] Li C (2015). Large stokes shift and high efficiency luminescent solar concentrator incorporated with CuInS_2_/ZnS quantum dots. Sci. Rep..

[33] Liu Z, Liu S, Wang K, Luo X (2010). Measurement and numerical studies of optical properties of YAG: Ce phosphor for white light-emitting diode packaging. Appl. Opt..

[34] Liu Z, Wang K, Luo X, Liu S (2010). Precise optical modeling of blue light-emitting diodes by Monte Carlo ray-tracing. Opt. Express..

[35] Luo H (2005). Analysis of high-power packages for phosphor-based white-light-emitting diodes. Appl. Phys. Lett..

[36] Kim JK (2005). Strongly enhanced phosphor efficiency in GaInN white light-emitting diodes using remote phosphor configuration and diffuse reflector cup. Japanese J. Appl. Phys..

[37] Tran NT, Shi FG (2008). Studies of phosphor concentration and thickness for phosphor-based white light-emitting diodes. J. Lightwave Technol..

